# Identification of *KISS1R* gene mutations in disorders of non‐obstructive azoospermia in the northeast population of China

**DOI:** 10.1002/jcla.23139

**Published:** 2019-12-10

**Authors:** Dongfeng Geng, Hongguo Zhang, Xiangyin Liu, Jia Fei, Yuting Jiang, Ruizhi Liu, Ruixue Wang, Guirong Zhang

**Affiliations:** ^1^ Center for Reproductive Medicine Center for Prenatal Diagnosis The First Hospital of Jilin University Changchun Jilin China; ^2^ Peking Medriv Academy of Genetics and Reproduction Beijing China

**Keywords:** *KISS1R*, male infertility, mutation, NOA, targeted exome sequencing

## Abstract

**Background:**

Non‐obstructive azoospermia (NOA), a serious phenotype of male spermatogenesis failure, is a multifactorial disease which is regulated by genetic, epigenetic, and environmental factors. Some gene structural variants have been demonstrated to be related to NOA. Loss‐of‐function mutations of *KISS1R* cause normosmic idiopathic hypogonadotropic hypogonadism (IHH) which result in azoospermia at the pre‐testicular level. The objective of this research was to investigate genetic variants of *KISS1R* in NOA patients.

**Methods:**

The entire coding region of 52 spermatogenesis‐associated genes (*KISS1R* included) was sequenced from 200 NOA patients. Mutation screening was performed to identify genetic variations of these genes by targeted exome sequencing. Sequencing data analysis was carried out by a series of bioinformatics tools. Candidate variants confirmation was performed by Sanger sequencing. Functional analysis of candidate variants was evaluated using SIFT and PolyPhen‐2.

**Results:**

Three heterozygous missense variants in *KISS1R* were identified in three patients, respectively. No deleterious variations in other candidate genes were found in the three patients. Two of these three variants, p.A211T and p.G186E, had been reported in the ExAC and dbSNP database, respectively, while the other variant p.A301D was novel. These variants were all predicted to be likely pathogenic by in silico analysis.

**Conclusion:**

Our study revealed three heterozygous missense variants in *KISS1R* which expanded the mutation spectrum of *KISS1R* in infertile men with NOA in the northeast of China.

## 1 INTRODUCTION

Azoospermia, which affects up to 1% of men in the general population,^1^ is a type of male infertility with a lack of spermatozoa in the ejaculate.^2^ Types of azoospermia include obstructive azoospermia (OA) and non‐obstructive azoospermia (NOA).^2^ Non‐obstructive azoospermia, a serious phenotype of male spermatogenesis failure, is a multifactorial disease which is regulated by genetic, epigenetic, and environmental factors. The genetic causes of NOA including Y‐chromosome microdeletions, karyotype abnormality and autosomal single‐gene or polygenic mutations or polymorphisms in multiple biological pathways are involved in the development of NOA.^3^ The prevalence of structural and numerical chromosomal abnormalities in the NOA population can up to 20%.^4^ Many other genetic causes of NOA remain unknown, although several genes have been reported based on an association between genetic variants of genes and NOA etiology—including PRM1, HSF2, KLHL10, SPATA16, AURKC, and ATM.^5^

The original purpose of our study was to investigate the contribution of genetic variations in some known causative genes associated with spermatogenesis to the development of NOA. Then in this study, we discovered and clarified genetic mutations of *KISS1R* gene in a population of infertile men with NOA.

The human *KISS1R* gene, located at 19p13.3, was initially identified as an orphan G protein‐coupled receptor (GPCR), and kisspeptin, a product of the *KISS1* gene, was its endogenous ligand.^6^ Loss‐of‐function mutations of *KISS1R* cause normosmic idiopathic hypogonadotropic hypogonadism (IHH) which result in azoospermia at the pre‐testicular level.^7^, ^8^ Kisspeptin binds to KISS1R stimulating GnRH release by hypothalamic neurons, leading to secretion of sexual steroids and pituitary gonadotropins, which in turn will play roles in producing the gametes.^9^ In the immature animals, precocious activation of the gonadotropic axis and pubertal development was able to induce by intermittent kisspeptin administration.^7^ In addition, animal studies suggested the targeted deletion of *KISS1R* led to hypogonadotropic hypogonadism, abnormal sexual maturation, and infertility.^6^, ^9^ Nevertheless, researches on kisspeptin and infertility are scarce.^9^ The understanding of genetic variations in *KISS1R* with NOA may lead to the use of *KISS1R* as a biomarker for diagnosis and treatments of male infertility.

## 2 MATERIALS AND METHODS

Our study consisted of 200 Chinese patients with idiopathic NOA. Mean age was 30.25 ± 5.24 years. All patients were diagnosed with NOA based on the following examination, including a detailed medical history, physical examination, hormone evaluation, semen analysis, Y‐chromosome microdeletions screening, and chromosome analysis. The exclusion criteria were as follows: (a) diagnosis with other clinical features; (b) chromosomal abnormalities that can be detected by cytogenetic analysis; (c) special diseases or history of chemical exposure that may affect spermatogenesis; and (d) Y‐chromosome microdeletions.

Mutation screening of genes associated with spermatogenesis was carried out by targeted exome sequencing as described previously.^10^ Genomic DNA was isolated from peripheral blood samples of all patients and subjected to exome capture using the in‐house targeted genes panel (Peking Medriv Academy of Genetics and Reproduction, Peking). Capture procedure was performed in solution that enriched the exonic sequences of 52 spermatogenesis‐associated genes (Table 1, *KISS1R* gene included) which obtained by reviewing the literature. Next‐generation sequencing was performed on the Illumina MiSeq sequencing platform (Illumina, Inc). Fastq sequence files were aligned against the human reference genome (NCBI build 37/hg19) with the Burrow‐Wheeler Aligner software (BWA version 0.7.12). Duplicated reads from the data sets were removed with Picard tools. Local realignment, recalibration, and variant calling were conducted with the Genome Analysis Tool Kit (https://software.broadinstitute.org/gatk/). Variants with minor allele frequencies >1% in the 1000 Genomes Project or in the dbSNP databases were excluded. Synonymous variants and variants in genes with unknown clinical phenotypes were filtered out. The remaining variants were evaluated for correlation with patient's phenotype. As our research objective, pathogenicity of the candidate variants was evaluated using SIFT (https://sift.bii.a-star.edu.sg/) and PolyPhen‐2 (https://genetics.bwh.harvard.edu/pph2/).

**Table 1:** Fifty‐two spermatogenesis‐associated genes included in the study

*AR*	*AURKC*	*CATSPER1*	*CCDC39*	*CFTR*	*CHD7*	*DNAAF1*	*DNAAF2*
*DNAAF3*	*DNAH1*	*DNAH11*	*DNAH5*	*DNAI1*	*DNAI2*	*DPY19L2*	*DYX1C1*
*ETV5*	*FGF8*	*FGFR1*	*GNRHR*	*HEATR2*	*HSF2*	*HYDIN*	*KAL1*
*KISS1R*	*LEP*	*LEPR*	*NANOS1*	*NELF*	*NR5A1*	*PLCZ1*	*PROK2*
*PROKR2*	*RHOXF1*	*RHOXF2*	*RSPH1*	*RSPH4A*	*RSPH9*	*SEPT12*	*SLC26A8*
*SOHLH2*	*SPATA16*	*SUN5*	*SYCE1*	*SYCP3*	*TAC3*	*TACR3*	*TEX11*
*USP26*	*WDR11*	*ZMYDN15*	*ZMYND10*				

The candidate pathogenic variants were confirmed by conventional PCR and Sanger sequencing (ABI 3730XL, BGI Genomics, Beijing Genomics Institute‐Shenzhen). PCR amplification was performed using the following primers (5′‐3′): exon4: TTTGCAGGGTGGCTGGGTG (F) and GGGTGCCGTGAAGGTGGTTAG (R); and exon5: GCCTTTCGTCTAACCACCTTCA (F) and CACTGCTCCCTGGCTTCTGC (R).

## 3 RESULTS

**Figure 1:**
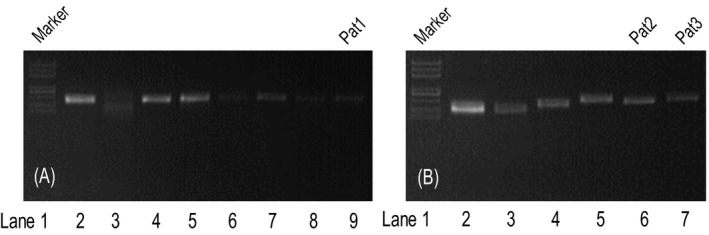
PCR amplification for the candidate pathogenic variants in the three patients. Lane 1: DNA marker is 5000, 3000, 2000, 1000, 750, 500, 250, and 100 bp from top to bottom. Lane 9 (A): Amplified products of Pat1. Lanes 6 and 7 (B): Amplified products of Pat2 and Pat3. Other lanes: Amplified products of other samples unrelated to this study

**Figure 2:**
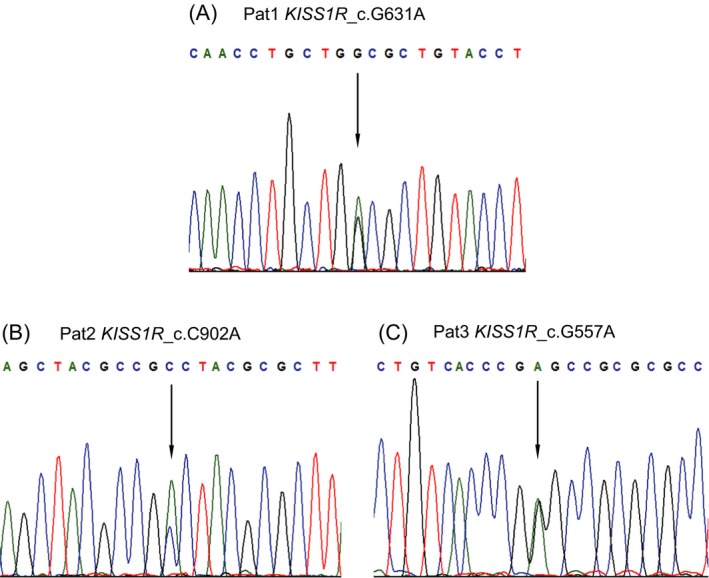
The three *KISS1R* missense variants in these three patients with NOA were confirmed by Sanger sequencing. The base mutation position is indicated by a black arrow. A, Heterozygous *KISS1R* c.G631A mutation was detected in pat1; B, Heterozygous *KISS1R* c.C902A mutation was detected in pat2; C, Heterozygous *KISS1R* c.G557A mutation was detected in pat3

Targeted exome sequencing was carried out in 200 patients with NOA, and spermatogenesis‐associated gene mutations were evaluated. To examine whether *KISS1R* genetic defects were associated with NOA, we focused on genetic variants in the exonic region of the *KISS1R* occurred in 200 patients with NOA. As a result, a total of 3 of 200 (1.50%) patients were found to have *KISS1R* variations, and we identified three *KISS1R* heterozygous missense variants in three cases, respectively (Table 2). No deleterious variations in other 51 candidate genes were found in the three patients. The p.A211T and p.G186E missense mutations had been reported in the ExAC and dbSNP database, respectively. The other variant p.A301D had never previously been reported, which was not found in the public databases including 1000 Genome Project, dbSNP, and ExAC database. According to SIFT software, the three variants were all predicted to be deleterious to the protein's function. The p.A211T and p.A301D variants were both possibly damaging, and the p.G186E variant was probably damaging according to PolyPhen‐2 (Table 3). We performed PCR and Sanger sequencing on the three patients and confirmed the three heterozygous missense mutations in these patients, respectively (Figure 1 and Figure 2). The relevant clinical and hormone data of these patients were summarized in Table 4. Color Doppler ultrasonography for scrotal of these three patients with missense variants revealed that Pat2 and Pat3 showed normal testicular volume, while Pat1 carrying p.A211T showed small testes in the scrotal sac. Hormone levels were normal or slightly lower than normal in the two patients with *KISS1R* mutations (Pat2 and Pat3). The hormonal level of the other one patient carrying p.A211T (Pat1) was obviously abnormal which exhibited high FSH level and a low T level.

**Table 2:** *KISS1R* variants identified in 200 patients diagnosed with NOA

No.	Exon	Position	cDNA mutation	Protein mutation	dbSNP135	Zygosity	Reported	Patients ID
1	4	919999	c.G631A	p.A211T	rs766694658	Hetero	Yes	Pat1
2	5	920453	c.C902A	p.A301D	/	Hetero	No	Pat2
3	4	919925	c.G557A	p.G186E	rs1281550153	Hetero	Yes	Pat3

Abbreviation: Hetero, heterozygous.

**Table 3:** Functional analysis of missense variants by bioinformatics software

No.	cDNA mutation	Protein mutation	Variant type	SIFT		PolyPhen‐2	
				Score	Prediction	Score	Prediction
1	c.G631A	p.A211T	Missense	0.02	Deleterious	0.688	Possibly damaging
2	c.C902A	p.A301D	Missense	0.02	Deleterious	0.599	Possibly damaging
3	c.G557A	p.G186E	Missense	0.00	Deleterious	0.910	Probably damaging

Note

SIFT: cut‐off score ≤0.05 for deleterious variants; PolyPhen‐2: score <0.15 for benign, 0.15‐0.85 for possibly damaging, and >0.85 for probably damaging.

**Table 4:** Clinical and hormone profiles of patients with NOA with *KISS1R* missense variants

Patients ID	Age (y)	FSH (mIU/mL)	LH (mIU/mL)	*T* (nmol/L)	E_2_ (pg/mL)	Testicular volume (Left/ Right, mL)
Pat1	42	21.9↑	7.3	9.4↓	23.6↓	11/11
Pat2	45	7.21	5.08	13.59	17.5↓	15/15
Pat3	21	2.95	3.09	17.21	37.4	15/15

Abbreviations: ↑: elevated; ↓: decreased; E_2_: estradiol (27.96‐155.92 pg/mL); FSH: Follicle‐stimulating hormone (1.5‐12.4 mIU/mL); LH: luteinising hormone (1.7‐8.6 mIU/mL); T: testosterone (9.9‐27.8 nmol/L).

## 4 DISCUSSION

In the present study, we have described three heterozygous missense mutations in the *KISS1R* gene in three patients with NOA. To our knowledge, this is the first reported heterozygous missense mutations in the *KISS1R* gene in the NOA population.

Idiopathic hypogonadotropic hypogonadism (IHH), which result in azoospermia at the pre‐testicular level, is characterized by a defect in the onset or maintenance of puberty caused by hypothalamic‐pituitary‐gonadal axis dysfunction with the absence of an organic lesion,^11^ and its clinical manifestation depends on the onset time. Males can be clinically manifested as absent or incomplete puberty, small penis, cryptorchidism, and infertility.^12^ In the past few years, IHH has classically been categorized as a single‐gene disease,^13^ but the phenotypic presentation of this disease and its genetic background are highly heterogeneous.^14^ A few genes that are involved in the pathogenesis of IHH have been identified at various sites, including *TAC3*, *TACR3*, *GnRHR*, *FGFR1*, *GNRH1*, *FGF8*, *KISS1,* and *KISS1R*.^11^ However, these genetic defects account for less than 30% of patients with IHH.^15^

In previous studies, *KISS1R* was one of the major genes that implicated in IHH in the autosomal‐recessive form.^16^ Mutations of this gene have been identified in less than 5% of patients with normosmic IHH.^12^ Up to now, at least 20 different mutations have been described in the literature, most of which were loss‐of‐function mutations and were found to have variable clinical manifestation.^12^, ^17^ *KISS1R* mutations which have been reported to be associated with IHH include point mutations, insertion, deletion, missense mutation, acceptor splice site mutation, and compound heterozygous mutation.^17^ According to the previous reports, there are no striking phenotype‐genotype relationships for *KISS1R* mutations. The inadequacies of *KISS1R* can manifest itself in different clinical entities. Complete loss‐of‐function mutation does not necessarily cause complete gonadotropic deficiency, and variable phenotypes could be present in patients carrying the same mutation.^18^

Current studies generally agree that IHH due to *KISS1R* mutations is transmitted as a recessive trait, although heterozygous mutations have been reported in the literature. Cerrato et al reported a heterozygous *KISS1R* mutation (c.A1079T/p.H360L) in a male IHH patient who had no pubertal development.^19^ Teles et al reported a heterozygous *KISS1R* variant (p.E252Q) in a male patient with sporadic normosmic IHH, who exhibited micropenis and cryptorchidism at birth, and no pubertal development.^20^ These studies indicated that heterozygous mutations in *KISS1R* could be also involved in the pathogenesis of IHH.

In the present study, we reported three heterozygous missense mutations in the *KISS1R* gene in three patients with NOA, in whom the typical IHH phenotype was not found but only infertility. The pathogenicity of these variants may be attributed to dominant‐negative effects. Chevrier et al have reported a heterozygous insertion in the intracellular domain of KISS1R that may lead to hypogonadotropic hypogonadism. The mutation disturbed the normal expression of the receptor at cell surface. The KISS1R bearing the heterozygous insertion exerts a dominant‐negative effect on the synthesis of the wild‐type KISS1R.^18^

There are some limitations in the report. Firstly, mutations of *KISS1R*, including heterozygous missense mutations, were reported in idiopathic hypogonadotropic hypogonadism, which results in NOA at pre‐testicular level. However, no related phenotype was found in these three patients with *KISS1R* heterozygous missense mutations in this report. So what is the possible mechanism that *KISS1R* mutations lead to spermatogenesis failure? In the report, we only make theoretical conjectures briefly. Secondly, functional study was not performed to demonstrate the pathogenicity of the three *KISS1R* variants. It should be demonstrated by proving the dominant‐negative effects of *KISS1R* mutations according to the method described in reference^18^ in our further study.

Although a definite inference about the consequences of the heterozygous *KISS1R* mutations has not yet made, we have presented three cases of NOA in three patients from northeast China due to three heterozygous missense mutations in the *KISS1R* gene. For better understanding of the correlations between *KISS1R* mutations and the relevant genetic background of patients with NOA, *KISS1R* gene detection should be recommended as a part of genetic screening of NOA patients. It is worth studying the related mechanism of spermatogenesis failure caused by *KISS1R* heterozygous mutation. We hope that our report will contribute to the ongoing genetic characterization of NOA.

## AUTHORS CONTRIBUTIONS

Ruixue Wang and Ruizhi Liu conceived and designed the experiments. Jia Fei, Yuting Jiang, and Guirong Zhang performed the experiments. Dongfeng Geng, Hongguo Zhang, Xiangyin Liu, and Yuting Jiang analyzed the data. Dongfeng Geng and Ruixue Wang wrote the paper.

## ETHICAL APPROVAL

This study was approved by the Medical Ethics Committee of the First Hospital of Jilin University (No. 2017‐404).

## CONSENT TO PARTICIPATE

Performed after obtaining written informed consent from the participants.

## References

[1] Nakamura S , Miyado M , Saito K , et al. Next‐generation sequencing for patients with non‐obstructive azoospermia: implications for significant roles of monogenic/oligogenic mutations. Andrology. 2017;5(4):824‐831. 28718531 10.1111/andr.12378

[2] Liu W , Gao X , Yan L , et al. Analysis of CDK2 mutations in Chinese men with non‐obstructive azoospermia who underwent testis biopsy. Reprod Biomed Online. 2018;36(3):356‐360. 29373224 10.1016/j.rbmo.2017.12.017

[3] Hu Z , Li Z , Yu J , et al. Association analysis identifies new risk loci for non‐obstructive azoospermia in Chinese men. Nat Commun. 2014;5:3857. 24852083 10.1038/ncomms4857

[4] Xie C , Chen X , Liu Y , Wu Z , Ping P . Multicenter study of genetic abnormalities associated with severe oligospermia and non‐obstructive azoospermia. J Int Med Res. 2018;46(1):107‐114. 28730893 10.1177/0300060517718771 PMC6011285

[5] Xu J , Jiang L , Yu W , et al. A novel functional variant in Wilms' Tumor 1 (WT1) is associated with idiopathic non‐obstructive azoospermia. Mol Reprod Dev. 2017;84(3):222‐228. 27990711 10.1002/mrd.22768

[6] Nimri R , Lebenthal Y , Lazar L , et al. A novel loss‐of‐function mutation in GPR54/KISS1R leads to hypogonadotropic hypogonadism in a highly consanguineous family. J Clin Endocrinol Metab. 2011;96(3):E536‐E545. 21193544 10.1210/jc.2010-1676

[7] Silveira LG , Noel SD , Silveira‐Neto AP , et al. Mutations of the KISS1 gene in disorders of puberty. J Clin Endocrinol Metab. 2010;95(5):2276‐2280. 20237166 10.1210/jc.2009-2421 PMC2869552

[8] Hamada AJ , Esteves SC , Agarwal A . A comprehensive review of genetics and genetic testing in azoospermia. Clinics (Sao Paulo). 2013;68(suppl 1):39‐60. 10.6061/clinics/2013(Sup01)06 PMC3583155 23503954

[9] Trevisan CM , Montagna E , de Oliveira R , et al. Kisspeptin/GPR54 System: what do we know about its role in human reproduction? Cell Physiol Biochem. 2018;49(4):1259‐1276. 30205368 10.1159/000493406

[10] Geng D , Yang X , Wang R , et al. A novel stopgain mutation c.G992A (p. W331X) in TACR3 gene was identified in nonobstructive azoospermia by targeted next‐generation sequencing. J Clin Lab Anal. 2019;33(3):e22700. 30390321 10.1002/jcla.22700 PMC6818563

[11] Demirbilek H , Ozbek MN , Demir K , et al. Normosmic idiopathic hypogonadotropic hypogonadism due to a novel homozygous nonsense c.C969A (p. Y323X) mutation in the KISS1R gene in three unrelated families. Clin Endocrinol (Oxf). 2015;82(3):429‐438. 25262569 10.1111/cen.12618

[12] Chelaghma N , Oyibo SO , Rajkanna J . Normosmic idiopathic hypogonadotrophic hypogonadism due to a rare KISS1R gene mutation. Endocrinol Diabetes Metab Case Rep. 2018;2018:1‐4. 10.1530/EDM-18-0028 PMC5911663 29692902

[13] Boehm U , Bouloux PM , Dattani MT , et al. Expert consensus document: European Consensus Statement on congenital hypogonadotropic hypogonadism–pathogenesis, diagnosis and treatment. Nat Rev Endocrinol. 2015;11(9):547‐564. 26194704 10.1038/nrendo.2015.112

[14] Cioppi F , Riera‐Escamilla A , Manilall A , et al. Genetics of ncHH: from a peculiar inheritance of a novel GNRHR mutation to a comprehensive review of the literature. Andrology. 2019;7(1):88‐101. 30575316 10.1111/andr.12563

[15] Bonomi M , Libri DV , Guizzardi F , et al. New understandings of the genetic basis of isolated idiopathic central hypogonadism. Asian J Androl. 2012;14(1):49‐56. 22138902 10.1038/aja.2011.68 PMC3735150

[16] Aoyama K , Mizuno H , Tanaka T , et al. Molecular genetic and clinical delineation of 22 patients with congenital hypogonadotropic hypogonadism. J Pediatr Endocrinol Metab. 2017;30(10):1111‐1118. 28915117 10.1515/jpem-2017-0035

[17] Nalbantoglu O , Arslan G , Koprulu O , Hazan F , Gursoy S , Ozkan B . Three siblings with idiopathic hypogonadotropic hypogonadism in a nonconsanguineous family: a novel kiss1r/gpr54 loss‐of‐function mutation. J Clin Res Pediatr Endocrinol. 2019;11(4):444‐448. 30905142 10.4274/jcrpe.galenos.2019.2018.0230 PMC6878343

[18] Chevrier L , de Brevern A , Hernandez E , et al. PRR repeats in the intracellular domain of KISS1R are important for its export to cell membrane. Mol Endocrinol. 2013;27(6):1004‐1014. 23608644 10.1210/me.2012-1386 PMC5415274

[19] Cerrato F , Shagoury J , Kralickova M , et al. Coding sequence analysis of GNRHR and GPR54 in patients with congenital and adult‐onset forms of hypogonadotropic hypogonadism. Eur J Endocrinol. 2006;155(suppl 1):S3‐S10. 17074994 10.1530/eje.1.02235

[20] Teles MG , Trarbach EB , Noel SD , et al. A novel homozygous splice acceptor site mutation of KISS1R in two siblings with normosmic isolated hypogonadotropic hypogonadism. Eur J Endocrinol. 2010;163(1):29‐34. 20371656 10.1530/EJE-10-0012

